# Identification of QTLs for Resistance to *Fusarium* Head Blight Using a Doubled Haploid Population Derived from Southeastern United States Soft Red Winter Wheat Varieties AGS 2060 and AGS 2035

**DOI:** 10.3390/genes11060699

**Published:** 2020-06-25

**Authors:** Alejandro Castro Aviles, Stephen Alan Harrison, Kelly Joseph Arceneaux, Gina Brown-Guidera, Richard Esten Mason, Niranjan Baisakh

**Affiliations:** 1 School of Plant, Environmental and Soil Sciences, Louisiana State University Agricultural Center, Baton Rouge, LA 70803, USA; castroaviles@wisc.edu (A.C.A.); sharrison@agcenter.lsu.edu (S.A.H.); KArceneaux@agcenter.lsu.edu (K.J.A.); 2 USDA-ARS Plant Science Research, Raleigh, NC 27695, USA; gbrowng@ncsu.edu; 3 Crop, Soil and Environmental Science, University of Arkansas, Fayetteville, AR 72701, USA; esten@uark.edu

**Keywords:** *Fusarium* head blight, QTL, markers, resistance, wheat

## Abstract

*Fusarium* head blight (FHB), caused primarily by the fungus *Fusarium graminearum*, is one of the most damaging diseases of wheat, causing significant loss of yield and quality worldwide. Warm and wet conditions during flowering, a lack of resistant wheat varieties, and high inoculum pressure from corn stubble contribute to frequent FHB epidemics in the southern United States. The soft red winter wheat variety AGS 2060 is moderately susceptible (as opposed to susceptible) to FHB and regularly found in pedigrees of resistant breeding lines. AGS 2060 does not carry any known resistance genes or quantitative trait loci (QTL). A QTL mapping study was conducted to determine the location and genetic effect of its resistance using a doubled haploid mapping population produced from a cross between wheat varieties AGS 2060 and AGS 2035 (FHB susceptible). The population was genotyped using the Illumina iSelect single nucleotide polymorphism (SNP) array for wheat and phenotyped in Baton Rouge and Winnsboro, Louisiana and Newport, Arkansas in 2018 and 2019. The effect of genotype was significant for *Fusarium* damaged kernels (FDK) and deoxynivalenol (DON) content across all locations and years, indicating genetic variation in the population. The study detected 13 QTLs (one each on chromosome 1A, 1B, 1D, 2A, 2B, 6A, 6B, 7A, and 7B, and two each on 5A and 5B) responsible for the reduction of FDK and/or DON. Of these, nine QTLs for FHB resistance were identified in Winnsboro, Louisiana, in 2019. QTLs on chromosomes 2A and 7A could be valuable sources of resistance to both DON and FDK over several environments and were likely the best candidates for use in marker-assisted selection. Consistently expressed QTLs on chromosomes 5A, 6B, and 7A were potentially newly identified sources of resistance to FHB in soft red winter wheat.

## 1. Introduction

Wheat is an important food staple in many countries, and wheat grain products play a very critical role in food security and the food processing industry [1]. Wheat provides ~19% of the food calories and 20% of the protein consumed worldwide [2]. It is cultivated on over 218 million hectares worldwide, the most of any crop, with a total production of over 750 million tons, second only to maize [3].

Soft red winter wheat (SRWW) (*Triticum aestivum;* 2n = 6× = 42; AABBDD genomes) is the common wheat that originated from the cross between the domesticated wild einkorn (*Triticum urartu*; 2n = 2× = 14, AA genome) and *Aegilops speltoides* (2n = 2× = 14, BB genome), resulting in the tetraploid emmer wheat (*Triticum turgidum ssp. Dococcum;* 2n = 4× = 28; AABB genome), which subsequently crossed with *Aegilops tauschii* (2n = 2× = 14, DD genome). Soft red winter wheats are grown in humid regions not suited for hard wheat production and are typically used to make flatbreads, cakes, cookies, crackers, pastries, and other crumbling products. In Louisiana and across the southeastern U.S., SRWW is a major crop, but total acreage fluctuates due to changing market prices, weather conditions, and disease pressure. The total area to plant wheat in Louisiana declined from 162,000 hectares in 2008 to only 16,190 hectares in 2016, which is the lowest production in the last 36 years [4]. Similarly, the value of wheat in Louisiana dropped from $120,000,000 in 2008 to $26,400,000 in 2010 [4]. One of the primary reasons for decreased production of wheat in recent years is the impact of *Fusarium* head blight (FHB or scab), a fungal disease caused primarily by *Fusarium graminearum*. FHB devastated wheat in Louisiana during the growing seasons from 2015 to 2017, which was attributed to the warm and rainy conditions during flowering, the lack of resistant wheat varieties, and the high acreage of corn that provided high inoculum pressure from the residual stubble [5].

FHB is one of the most damaging diseases in wheat growing areas worldwide [6]. Severe FHB epidemics may occur when susceptible varieties encounter the pathogen inoculum under warm and humid conditions during flowering, and the early stages of kernel development are favorable for FHB infection [7,8]. The FHB epidemics between 1998 and 2001 in the Great Plains resulted in a cumulative direct economic loss of $1.074 billion in wheat and barley [9]. FHB infections cause significant losses in yield and grain quality through shriveled kernels [7]. The infected grain is also contaminated with a mycotoxin group called trichothecenes, the most common being deoxynivalenol (DON). DON is produced before harvest and thus cannot be completely avoided. Grain contaminated with DON may be unsuitable for food or feed use due to serious concerns to human and animal health [10]. 

FHB epidemics have become more frequent and more severe due to agronomic practices, such as conservation tillage that retain inoculum-containing stubble, causing significant economic losses [11]. The use of resistant cultivars, coupled with cultural practices, such as crop rotation, conventional tillage, and fungicide application, can effectively reduce losses caused by the disease. Nevertheless, the deployment of disease-resistant cultivars is the most economical and sustainable method of controlling FHB in wheat [12]. A comparison of DON reduction resulting from different agronomic practices has shown that planting resistant wheat cultivars result in the largest decrease in DON concentration [13]. 

A large variation exists in genetic sources for FHB resistance in the wheat gene pool. However, the best regionally adapted and high yielding cultivars often lack desirable resistance to FHB [14]. Breeders rely on a small number of important genes with moderate effects [15]. There is a need to discover and incorporate new sources of resistance into new breeding lines.

FHB resistance is a polygenic trait with moderate heritability and is highly influenced by the environment. Thus, screening for resistance in inoculated nurseries using conventional phenotyping can be difficult, time-consuming, and cost-intensive [10,14,16]. Marker-assisted selection of genes or quantitative trait loci (QTLs) can help overcome limitations associated with conventional phenotypic evaluations of genetically complex traits, such as FHB resistance [17]. 

Over 200 QTLs, including seven designated genes *Fhb1* to *Fhb7*, conferring varying levels of FHB resistance have been reported [16,18]. QTLs for FHB resistance have been found on all wheat chromosomes, where 22 QTLs have been detected in more than one mapping population with the most repeatable QTL on chromosomes 3BS (*Fhb1*; syn. *Qfhs.ndsu-3BS*), 6BS (*Fhb2*), and 5AS (*Qfhs.ifa-5A*) [14]. Cativelli et al. [19] mapped an FHB resistance gene on chromosome 7D of the cultivar Catbird in 2013. Using QTLs reported in 30 different mapping populations, Löffler et al. [20] identified 19 meta-QTLs (MQTLs) on 12 chromosomes. A meta-analysis of 209 QTLs, which comprised of 48% QTLs from Asia and only 14% from North and South American sources, from 45 mapping studies, has classified the QTLs into 43 clusters with 15 on both A and B genome and 13 on D genome [21]. The authors reclassified the 19 MQTLs identified by Löffler et al. [20] into 18 because the two MQTLs on chromosome 5A were within 20 cM. 

Most sources of FHB resistance described until recently are from spring wheat lines of Asian and South American descent. Moderately susceptible lines have also been shown to contain valuable sources of resistance, such as the Chinese spring wheat cultivar Sumai 3, which contains *Fhb1* and *Fhb2* and is developed from two moderately susceptible parents—Funo and Taiwanxiaomai [22,23]. Thus far, *Fhb1*, first reported in Sumai3 [10,24] and subsequently in different genetic backgrounds [14,25,26,27], is the most stable major QTL that provides resistance to a broad spectrum of *Fusarium* species [28]. Diagnostic kompetitive allele specific PCR (KASP) markers have been developed for *Fhb1* and are being used in wheat breeding programs worldwide [28,29,30,31].

Due to the linkage drag of undesirable traits that come with *Fhb1* source cultivar, Sumai 3, and other exotic sources of resistance, incorporation of these sources into regional SRWW breeding programs has not been very successful. There are, however, adapted SRWW cultivars, such as Ernie, Truman, Bess, Massey, Roane, Tribute, Jamestown, Freedom, and Goldfield, that possess moderate levels of FHB resistance [32]. Jamestown is currently used as the main source of FHB resistance in SRWW, and it serves as a moderately resistant check in the southern uniform winter wheat scab nursery. It harbors a QTL on chromosome 1B, which accounts for 12.7% to 13.3% and 26.1% of the variation for DON accumulation and FHB severity, respectively [33]. Neuse is another moderately FHB-resistant SRWW cultivar with three QTLs, one each on chromosome 1A (for reduced incidence, severity, *Fusarium* damaged kernels (FDK), and DON), chromosome 4A (for reduced FDK and DON with 19.5% of the phenotypic variation), and chromosome 6A (for significantly reduced incidence and severity) [34]. Bess is an SRWW variety that has been used as a resistant check in the U.S. winter wheat southern nursery since 2007. It possesses two QTLs (one each on chromosomes 2B and 3B) associated with reduced severity, FDK, and DON. Multiple QTLs/genes from these sources, such as *Fhb_5A* (Ernie), *Fhb_3B* (Massey), *Fhb_1B* (Jamestown), *Fhb_1A* (Neuse), *Fhb_4A* (Neuse), *Fhb_6A* (Neuse), *Fhb_2B* (Bess), and *Fhb_3B* (Bess), are currently being used for marker-assisted FHB resistance breeding programs in the southeastern US. Many winter wheat genotypes with mapped resistance QTLs are of European descent [35]. Pyramiding of the resistance QTLs is the most practical approach for improving FHB resistance in locally adapted cultivars. Varieties, such as AGS 2060, which are intermediate in reaction to FHB but do not contain known major resistance genes, such as *Fhb1*, offer the opportunity to identify novel genes for FHB resistance that can be pyramided for effective, durable resistance. The objective of the present study was to identify new QTLs in two SRWW varieties that could be integrated into the SRWW breeding program. Here, we reported the identification of QTLs for FHB resistance using a doubled haploid (DH) mapping population derived from a cross between AGS 2060 and AGS 2035.

## 2. Materials and Methods

### 2.1. Mapping Population

The mapping population consisted of doubled haploid (DH) lines developed using standard corn pollen methodology [36,37] from F_1_ seeds of the cross between AGS 2060 and AGS 2035 (PVP200900420), a highly FHB susceptible variety. AGS 2060 is a soft red winter wheat variety released by the Louisiana State University in 2004 (PVP 200800412). It is moderately susceptible to FHB but found in pedigrees of several resistant breeding lines and cultivars. AGS 2035 (PVP200900420), a highly FHB susceptible SRWW variety, was developed by the University of Georgia Agricultural Experiment Station in Griffin, Griffin, GA, USA.

### 2.2. Disease Screening

One hundred ninety-two DH lines and the parents were grown as two-row plots in a randomized complete block design with two replications in misted and inoculated nurseries at Ben Hur Research Farm in Baton Rouge, Louisiana in 2017 and 2018 (designated as BR17 and BR18), Macon Ridge Research Station in Winnsboro, Louisiana in 2018 and 2019 (designated as WN18 and WN19), and Newport Research and Extension Center in Newport, Arkansas in 2018 (designated as NP18). Each row measured ~100 cm with 38 cm spacing between the rows. Plots were planted using a Hege^®^ 90 magazine planter system with a Trimble^®^ RTK system GPS (Wintersteiger Inc., Salt Lake City, UT, USA) guided tractor using autosteer to maintain uniform row spacing. The plots received standard production practices recommended for wheat in Louisiana [38]. The plots were inoculated twice in early March when the 2 nd node was visible, and mid-March with *F. graminearum* infected corn kernels that were evenly spread (5 g/row) across the nursery. Sterilized corn kernels were inoculated with mixtures of isolates of DON genotype *F. graminearum* collected from locally grown infected wheat fields using the standard method described in the international scab nursery consortium (https://scabusa.org/pdfs/ptt/Gilbert_Field-Screening.pdf). The sprinkler system misted the nursery twice nightly for 20 min each in Baton Rouge and four times for 15 min each in Winnsboro to provide favorable moist conditions for fungal development. Sprinklers were maintained from the second node through mid-dough stages for optimal fungal growth during critical disease development stages. In Newport, mist irrigation, following the spread of inoculum, was set up every sixth row, covering the entire disease nursery. Mist irrigation commenced when perithecia were observed on the maize inoculum to provide optimal conditions for FHB infection and spread throughout April and May. The duration of misting was adjusted according to the weather data on available precipitation and dew point.

### 2.3. Phenotypic Data and Statistical Analysis

Data were collected for disease severity and incidence, 0–9 rating, and heading date or relative maturity (where evaluating heading dates frequently was not feasible). Disease incidence is a percentage estimate of the total number of heads, which are infected with the disease in a row or plot. Disease severity is a percentage estimate of the total wheat head surface area infected with the disease of those heads showing infection. Disease index is a combination of incidence and severity obtained by multiplying both values and expressed in percentage, an estimate of the total proportion of heads showing infection. The 0–9 rating represented visual evaluation of disease severity and index, where 0 denotes completely disease-free florets, and 9 indicates that >90% florets were infected. An increase of 1 unit roughly corresponded to a 10% increase in the proportion of diseased florets. The heading date was recorded as the day of the year when 50% of the heads in a plot completely emerged, or every head in a line was emerged by 50%. Each line was rated once at the adult stage when disease symptoms were visible on the plants in Winnsboro and Baton Rouge. Field ratings were recorded on several days over a span of three weeks, between 14 to 21 d after the heading of each line. The field trial in Arkansas was non-replicated, with ratings taken only on the FHB disease severity index.

At maturity, when the seeds were dry, plots were individually harvested with rice knives and threshed in a Vogel thresher using minimal airflow to retain shriveled and scabby kernels. Collected seeds were then hand-screened and cleaned prior to visually rating the percentage of *Fusarium* damaged kernels (FDK). A standard set of FDK dilution samples was used to calibrate visual estimates of FDK. FHB can cause discoloration or bleaching in the head, which could be confused with plant senescence, and disease progression is somewhat dependent on days past heading. Therefore, days to the heading or relative maturity were rated in each test to evaluate if ratings were being skewed by maturity differences. Seed samples from each line were submitted to the Mycotoxin Diagnostic Laboratory at the University of Minnesota, St. Paul, Minnesota, for the quantification of deoxynivalenol (DON) by gas chromatography-mass spectrometry.

### 2.4. Statistical Analysis

The analysis of variance (ANOVA) tests were performed using the general linear model in SAS ver. 9.2 (SAS Institute, Inc., Cary, NC, USA) for DON, FDK, disease index, incidence, severity, and the 0–9 FHB rating. Separate ANOVA tests were performed for each environment (location and year), except Newport, Arkansas, where there was only one entry of each line. Environments evaluated included BR17, BR18, WN18, and WN19. Correlations between all disease traits were determined by Pearson’s correlation test using SAS ver. 9.2. Broad-sense heritability (*H^2^*) was calculated for FDK and DON based on a plot mean basis for each trait across locations/years using variance components, as described earlier [39], using ICI mapping software ver. 4.2 [40] with the analysis of variance for the multi-environmental trials option.

### 2.5. Genotyping and Marker Data Processing

Leaf tissue (~2 cm) of each DH seedling was collected during the two-leaf stage of development (7 d after germination) from the growing points in 1.2 mL plastic storage tubes (Qiagen Inc., Valencia, CA, USA). The tubes containing collected tissue were covered with cheesecloth and inverted over silica in sealed plastic food containers to remove moisture. Genomic DNA was isolated from dried leaf tissue using the Mag-Bind^®^ Plant DNA Plus Kit (Omega Bio-tek, Norcross, GA, USA) and quantified using Quant-iT™ PicoGreenTM dsDNA Assay Kit (ThermoFisher Scientific, Carlsbad, CA, USA).

Genotyping of the DH population was performed with the publicly available Illumina iSelect single nucleotide polymorphism (SNP) array that contained 81,578 SNP markers distributed across all wheat chromosomes [41]. The assay was performed using protocols developed by the International Wheat SNP Consortium [42]. The genotypic data were downloaded using Illumina’s Genome Studio software and converted to an excel file. Letters distinguishing genotypes were converted to a number, where the parental homozygous alleles were assigned as “0” or “2” and heterozygotes as “1”. 

Marker polymorphism between the parents was low, so non-polymorphic SNPs were discarded. Bi-allelic SNPs with minor allele frequency less than 5% and more than 50% missing values were eliminated. Because DH lines are considered homozygous, heterozygote alleles (9.07%) were designated as missing data. The physical location of the SNPs was determined by aligning corresponding read sequences against the *Triticum aestivum* reference genome [43] using BWA ver 0.7.10 [44]. SNPs in close physical proximity (within 120 nt) and with more missing information (1.84%) were filtered out to retain evenly distributed unique markers for linkage mapping. 

### 2.6. Linkage and QTL Mapping

ICIMapping software ver. 4.2 [40] was used for both linkage analysis and QTL mapping. For the development of the linkage map, the multipoint linkage analysis function was used. Linkage mapping was performed with the maximum recombination frequency set at 0.4, and the Kosambi mapping function was used to calculate the genetic distance in centiMorgans (cM) between markers. A logarithm of odd (LOD) value ≥ 3 was set as the threshold for markers to be considered in the same linkage group.

FDK and DON were selected for QTL analysis as these traits are highly correlated and considered the most important as more accurate phenotypes of FHB resistance. FDK and DON data were averaged over years across locations and averaged over all locations and years for a grand overall mean. Thus, QTL analysis was performed for a total of 16 models, comprised of FDK and DON for the five environments individually (BR17, BR18, WN18, WN19, and NP18), averaged for Winnsboro, averaged for Baton Rouge, and averaged over all locations and years. 

QTL analysis was conducted using composite interval mapping (CIM) and a walking speed of 0.1 cM with 1000 iterations. A LOD value of 2.5 was set to call a QTL significant (*p* = 0.05). Information on the peak, position, LOD, flanking markers, confidence interval, phenotypic variance explained (PVE), and additive (A) mode of gene action was collected for each QTL. The nomenclature used to name the QTLs was “Q”, followed by “F” for *Fusarium*, the abbreviation for ratings “don”, “fdk”, and “d&f” for both, the institution where it was detected “LSU”, and chromosome number (1A, 1B, 1D–7D).

## 3. Results 

### 3.1. Disease Ratings

FHB disease pressure was high in the misted and inoculated nurseries with few DH lines, showing high levels of resistance (Table 1, Table 2, Table 3, Table 4 and Table 5). This was partly due to the high levels of disease inoculum, optimum conditions for the disease development, and no known QTLs for resistance in the parents of the population. In every trait rated for FHB resistance, AGS 2060 had lower means than AGS 2035, except for DON content in WN19 (Table 4) and NP18 (Table 5). The population mean of the DHs was within the parental limits for most traits, except DON and FDK in NP18 and DON in WN18 and WN19, where the population mean was higher than both parents. There were a few outliers with extremely high levels of DON content in each environment (Table 1, Table 2, Table 3, Table 4 and Table 5).

There were significant differences among the DH lines for all FHB traits in all environments (Table 1, Table 2, Table 3, Table 4 and Table 5). The average DON content ranged from 10.1 to 11.4 ppm, but it was much higher in Arkansas (27 ppm). FDK, index, and severity were also higher in Arkansas. The coefficients of variation (CV) for DON were higher than for other traits but were still within reasonable ranges for field trials.

FDK was significantly correlated to DON in each environment, with values ranging from 0.532 (WN19) to 0.733 (WN18) (Supplementary Tables S1–S5). Relative maturity or heading date was not highly correlated with FHB ratings, except in WN19, where relative maturity was the most closely correlated trait with FDK (0.496) and DON (0.697) (Supplementary Table S4). Late-heading lines generally had higher DON at Winnsboro, but there was little relationship between the traits in Baton Rouge (Supplementary Tables S1 and S2). 

**Figure 1:**
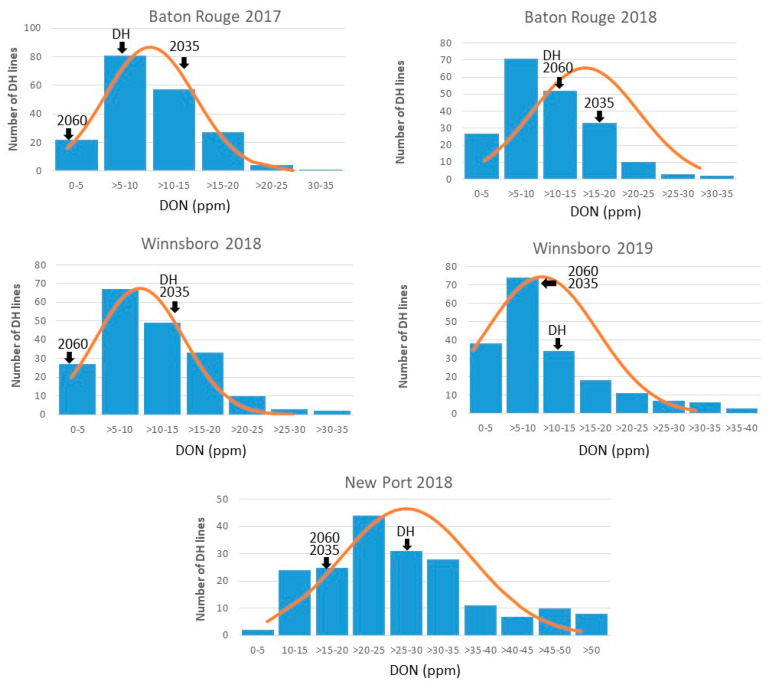
Phenotypic distribution of deoxynivalenol (DON) concentration for field trials in Baton Rouge, LA during 2017 and 2018; Winnsboro, LA during 2018 and 2019; Newport, AR during 2018. Parental and population means are marked by arrows. 2060, 2035, and DH represent mean values for AGS 2060, AGS 2035, and DH population, respectively. The normality of the data was tested using the Shapiro–Wilk test in R. Distribution was considered near-normal when *p*-values were less than but close to 0.05.

DON content showed near-normal distribution in each location (Figure 1), suggesting that DON was a quantitative trait and not controlled by major genes that would otherwise result in distinct classes. In each location, the highest number of lines had low DON in the range of 5–10 ppm, except for NP18, where the highest number of lines (32) were in the range of 25–30 ppm (Figure 1).

Broad sense heritability (*H^2^*), calculated across locations, for FDK and DON was 0.59 and 0.33, respectively. On the other hand, *H^2^*, calculated across years, for FDK and DON were 0.53 and 0.47, respectively (Supplementary Table S6). This indicated a larger genetic effect for FDK compared to DON.

### 3.2. Linkage and QTL Mapping

Of the 81,578 potential SNP markers in the Illumina iSelect wheat 90K SNP array, only 2011 biallelic SNPs were selected for linkage/QTL mapping after filtering. Linkage analysis resulted in 21 linkage groups, each corresponding to one chromosome of wheat. The linkage map covered 3746.80 cM of the wheat genome, with an average of 1.86 cM between markers. The linkage groups had between six (chromosome 3D) and 367 (chromosome 2B) SNPs (Supplementary Table S7). In general, the D chromosomes had lower marker density compared to A and B chromosomes.

A total of 13 QTLs distributed over 11 chromosomes were found to have a significant effect on FDK and/or DON in the population with LOD values between 2.51 and 5.54 (Table 6; Supplementary Figure S1). Chromosomes 5A and 5B harbored two QTLs each, and one QTL each was identified on chromosomes 1A, 1B, 1D, 2A, 2B 4B, 4D, 6A, 6B, and 7B. The percentage of phenotypic variation explained for a trait in a specific environment was highest (5.89%) for the QTL on chromosome 5A for DON in Winnsboro.

Four of the 13 QTLs had a significant effect on both DON and FDK, six QTLs on DON only, and four on FDK only (Table 6). Most QTLs were detected in multiple locations or years, and only four QTLs were detected in just one environment or condition. The highest number of QTLs (11) were detected in WN19, while 3, 4, 4, and 4 QTLs were detected in BR17, WN18, NP18, and BR18, respectively. 

Of the four QTLs associated with low DON and FDK, *QFd&f.LSU-2A* on chromosome 2A was the most consistent QTL detected in five different models. The QTL for all five models was genetically within 0.2 cM of each other. It was also the only QTL detected for mean DON across all environments. The QTL was detected with the highest LOD value (5.54 for WN average DON) and the third-highest PVE (4.12% for overall DON average). *QFd&f.LSU-1B* on chromosome 1B was another important QTL for both FDK and DON detected for three models within 1.5 cM of each other. The highest LOD value for this QTL reached at 4.82 (for WN19 FDK), and the highest PVE was 2.66% (for WN 2019 FDK). The favorable alleles for these two QTLs came from the resistant parent AGS2060. The QTL on chromosome 7A (*QFd&f.LSU-7A*) was also consistently expressed in four models, mapped within 5 cM of each other. It was detected in 2 locations and 3 years for DON (BR17, WN18, WN19) and 2 years for FDK (BR17, WN19). However, the alleles for low FDK or DON were contributed by AGS2035, except for low DON in BR18 that was contributed by AGS2035. *QFd&f.LSU-2B* on chromosome 2B was expressed for both DON and FDK in BR17, but for only DON in WN19. In this case, AGS2035 provided the favorable alleles for low DON and FDK in Baton Rouge and Winnsboro.

Two QTLs, 110 cM apart, were detected on chromosome 5A. The QTL *QFfdk.LSU-5A* for resistance to FDK was detected in two models, 9 cM apart, which had a LOD value of 3.2 for NP18. The other QTL *QFdon.LSU-5A* for DON was detected in three models within a distance of 1 cM, with the peaks at 40.68 and 41.19 cM. This QTL recorded the highest LOD (7.82) in WN19 and the highest PVE (5.89%) for the WN average.

Chromosome 6A contained one QTL, *QFdon.LSU-6A,* for DON resistance in both years in Winnsboro, Louisiana. *QFdon.LSU-6A* was significant for DON in 3 models, including 2 locations over 2 separate years with the PVE of 3.85% (WN18), 1.82% (WN19), and 1.81 (NP18). A QTL on chromosome 5B (*QFfdk.LSU-5B*) was detected for only FDK resistance in Baton Rouge with a PVE up to (1.83%) and a LOD value of 3.99.

## 4. Discussion

Efforts have intensified to identify sources of FHB resistance in locally adapted wheat cultivars that provide resistance without negatively affecting selection for other desirable traits. The present study identified novel sources of resistance in a Louisiana bred soft red winter wheat cultivar AGS 2060. 

### 4.1. Disease Ratings

Segregation of most complex traits, such as disease resistance traits, in a population, does not fit to simple Mendelian ratios because the traits are quantitative and controlled by multiple loci [45]. Quantitative resistance reduces the severity of disease by slowing the development of an epidemic without preventing them completely. Results of disease rating (Table 1, Table 2, Table 3, Table 4 and Table 5) and QTL analysis (Table 6) confirmed that FHB resistance in this population was a quantitative trait caused by the synergistic expression of several genes with small individual effects on the trait. This was apparent from the normal distribution of the traits in the population, where FHB ratings did not segregate into distinct classes expected with major genes (Figure 1), for example, populations segregating for *Fhb1*. Few DH lines showed potential for selection with high levels of resistance. For example, LA12016DHA-79 was the only line to average less than 9 ppm DON in each environment, which was excellent, given the intensity of disease pressure in the misted nurseries. Over half the lines averaged over 13.5 ppm DON across all environments (Supplementary Table S8). Neither parent had known major QTLs or moderately resistant reaction type, so it was expected that average DON content and FDK would be high under inoculated conditions.

The average DON content across all environments was 9.5 ppm for AGS 2060 and 11.8 ppm for AGS 2035. AGS 2060 had lower DON content in three of five environments. Coefficients of variation for DON among the DH lines were the highest of all traits measured, ranging between 36.4% and 49.7%. There was some variation in genotypic ranking for DON content over environments. Line LA12016DHB-8, for example, had a DON content of 5.3 ppm in BR18 and 70.1 ppm in NP18. The mean DON content of DHs was intermediate to slightly higher than the means of parents, except for NP18, where the population mean (27 ppm) was higher than that of either parent (AGS 2060, 18.4 ppm; AGS 2035, 15.6 ppm). This could be due to a lack of replication, weather pattern, or variation in the heading date in Arkansas. Average FDK content for AGS 2060 and AGS2035 was 24.4 and 44.4, respectively, suggesting that the resistance mechanism in AGS2060 could be for FDK as opposed to DON. DON content and FDK were consistently correlated across environments with significant (Pr ≤ 0.0001) coefficients of correlations, which reached as high as 0.733 in WN18. A strong correlation (0.819; Pr = 0.001) between FDK and DON has been previously reported [46].

### 4.2. Linkage and QTL Mapping

QTL mapping is an effective approach for studying the genetics of complex traits, such as disease resistance, and determine the location and effect of disease resistance loci in the genome [45]. It also provides an opportunity for QTL-assisted breeding for disease resistance and positional cloning of partial resistance genes. It is an initial step to developing trait-specific diagnostic markers for their use in the selection and advancement of lines in breeding programs. The success of QTL mapping depends on adequate phenotypic data and a sufficient number of polymorphic markers across the genome [47]. The level of polymorphism between AGS 2060 and AGS 2035 was lower than is commonly reported for wheat. This might be due to the fact that both varieties were soft red winter wheat lines developed for the southeastern U.S., and regional breeding programs have a high germplasm interchange, leading to lines sharing common ancestors in their pedigree.

Some of the QTLs identified in the DH mapping population in this study co-localized with known QTLs reported for FHB resistance in different populations. Giancaspro et al. [48] identified a QTL on chromosome 2A that explained 12% phenotypic variation for resistance to both FHB incidence and severity in a population derived from the cross between a resistant Chinese breeding line and a durum susceptible cultivar. RAC875_rep_c78744_228 (IWB63138), the closest marker to this QTL, at 31,957,675–31,957,775 bp on chromosome 2A, was physically located between the SNP markers—BS00023052_51 (3,111,127 bp) and BS00067792_51 (319,045,668 bp)—the two markers flanking the QTL *QFd&f.LSU-2A* for both FDK and DON identified in several environments in the present study. This important, well-studied source of resistance co-localizes with *WAK2* and *WheatPME1* genes on the same region of the short arm of chromosome 2A [48]. *PME* is suggested to be responsive to *Fusarium* infection by affecting the degree of cell wall methyl-esterification and pectin content. Wall-associated receptor kinase (WAK) allows plant cells to respond to their external environment due to an extracellular region associated with the pectin fraction of the cell wall. Interaction of *WAK2* and *PME1* in response to *Fusarium* infection has been described in durum wheat [49]. In addition, the *Rar1* gene, reported to be involved in *YrSu*-mediated stripe rust defense in wheat [40], was identified at BS00067792_51, the closest marker to *QFd&f.LSU-2A* (Supplementary Table S9). Accumulating evidence also suggested the role of folate-biopterin and gliadin genes, linked to this QTL, in disease resistance in plants. 

The QTL *QFfdk.LSU-5A* on chromosome 5A, detected in the present study in two models, is flanked by the markers BS00062907_51 and Ku_c12469_837 located at 33.01 Mbp and 596.5 Mbp, respectively. A QTL *Qfhs.ifa*-*5A* has been reported on chromosome 5A that predominantly provides type 1 resistance to FHB by preventing fungal entry [50]. *Qfhs.ifa*-*5A* is delimited by the markers—*barc186* (46.6 Mbp) and *wmc805* (364.4 Mbp)—which are 1.6 cM apart from each other [51] and within the physical range of *QFfdk.LSU-5A,* as identified in the present study. This suggests that *QFfdk.LSU-5A* and *Qfhs.ifa*-*5A* could possibly be the same QTL. However, the QTL *QFdon.LSU-5A* identified in this study exclusively for DON with the highest LOD (7.82) did not co-localize with any published QTLs and, therefore, could provide a new source of resistance. The pentatricopeptide repeat domain-containing protein, a gene linked to the closest marker Tdurum_contig11173_79 of the QTL, was significantly expressed in a near-isogenic line carrying the *Fhb1* gene [52] and highly expressed in spike at the FHB resistance QTL at *Traes_6DS_A9E719CC8* [23]. The protein is believed to be a key player in the signal transduction mechanism, leading to FHB resistance. Another gene, peptidyl-prolyl cis-trans isomerase (*PPI*) NIMA-interacting 4, linked to the marker, is implicated as a chaperone in cell death and stress tolerance [53]. PPI proteins show antifungal properties like PR proteins. The expression of a *PPI* is significantly upregulated (0.71 fold) in the resistant wheat cultivar Dream at 32 h after inoculation with *F. graminearum* [54]. 

The gene *Fhb3* for FHB resistance on the distal region of the short arm of chromosome 7A, which is selected by the linked marker BE585744 located at 233.3 Mbp [55], was different from the QTL *QFd&f.LSU-7A* identified in our study (Table 6). The proximal marker, IAAV822, flanks the QTL co-localized with a WD40 repeat-containing protein that mediates regulatory pathways in plant immunity [56]. In wheat, a WD40 homolog has been shown to be associated with resistance to necrotrophic pathogens [57] and drought resistance [58]. A leucine rich repeat (LRR) receptor-like serine/threonine-protein kinase HSL2 has been identified to be close to the distal flanking marker Tdurum_contig42424_291. Receptor-like kinases are involved in signaling pathways in plant defense response. In wheat, LRR kinases have been induced in response to infection by *Puccinia triticina* [59] and *Rhizoctonia cerealis* [60]. A serine/threonine-protein kinase LRK10 has also been associated with the stripe rust resistance locus *Lr10* [61]. 

Several wheat lines, including Seri 82, Fundulea 201R, Alondra, and Lynx, carry an FHB resistance QTL in the T1BL.1RS translocation region of chromosome 1B. The QTL discovered in variety Seri 82 is closely linked to *Xgwm153* marker [62] located at 628,826,245–628,826,264 bp, which is in physical proximity to the marker BS00064162_51 (601,007,290–601,007,313 bp), closest to the QTL *QFd&f.LSU-1B* on repeat region detected in AGS 2060 in our study.

Meta-QTL study has shown the entire length of chromosome 2B comprising of QTL intervals for FHB resistance [14]. The marker RAC875_c35399_497, closest to *QFd&f.LSU-2B*, on chromosome 2B, detected for both DON and FDK in Baton Rouge and Winnsboro in our study is physically located at 740,802,332 bp, which is located proximally to the marker *Xwmc149*, closest to the QTL at 779,109,515-779,109,536 bp, reported by Gilsinger et al. [63], suggesting that the two QTLs are most likely the same. In addition to an uncharacterized protein, an avenin-like protein (ALP-1) has been identified underlying the *QFd&f.LSU-2B*. Wheat *ALP* genes are upregulated in response to FHB inoculation in the embryo and (sub)aleurone layer cells, and the recombinant proteins show significant antifungal activity against *Fusarium graminearum* by virtue of their protease activity, inhibiting the infection process-related pathogen protein—beta-glucosidase [64]. Similarly, ATP-dependent DNA helicase, which may play an important role in stress tolerance in plants [65], has been found significantly abundant in wheat grains as a consequence of FHB inoculation [66]. 

Bourdoncle and Ohm [67] used recombinant inbred lines and discovered a QTL for resistance to FHB severity on chromosome 5B, which explained 7.1% of the overall phenotypic variation. The simple sequence repeat (SSR) marker *xbarc59* closest to this QTL is physically located between the SNP markers BS00079166_51 and CAP11_c7700_247, flanking the QTL *QFdon.LSU-5B*, detected in the DH mapping population of the present study, suggesting that both QTLs are the same. The closest marker CAP11_c7700_247 has been linked to E3 ubiquitin ligase (RNF170) and early salt and cold-acclimation-induced protein (Esi2) and two uncharacterized proteins. The signal transduction phosphoprotein E3 ubiquitin ligase has been shown to be abundantly accumulated in wheat spikes after *F. graminearum* inoculation [68]. More importantly, an E3 ubiquitin ligase has been shown to interact with the pore-forming toxin (*PFT*) gene at the *Fhb1* locus to confer resistance to FHB [69]. 

Chromosome 6A contained the QTL *QFdon.LSU-6A* for DON resistance. A QTL for FHB resistance has also been previously reported on chromosome 6A [14,70]. The marker *Xbarc107* linked to the QTL identified by Schmolke et al. [67] is physically located at 495,108,347–495,108,372 bp, which is physically near to marker wsnp_CAP11_c1137_665073 (542,232,102 bp), closest to *QFdon.LSU-6A* peak. Geranylgeranyl hydrogenase (*Ggh*), the only gene within 20 kb of the marker wsnp_CAP11_c1137_665073, closest to the QTL, has been shown to be regulated by pathogenic fungus *Taphrina deformans* in peach, suggesting its role in plant defense response [71].

Chromosome 6B contains *Fhb2*, one of the most reliable FHB resistance genes, which is flanked by the SSR marker *gwm644* at 217,955,423–217,955,440 bp [14]. Genomic regions for FHB resistance overlapping *Fhb2* have been detected in the background of Sumai 3 and Blackbird [14]. The QTL identified in Sumai 3 has explained up to 4.9% of the phenotypic variation [25]. Another QTL on chromosome 6B, explaining 9.2% of the variation, is flanked by the SSR marker *xbarc101*, which is physically (216,038,675–216,038,696 bp) very close to *gwm644* flanking *Fhb2*. The QTL *QFfdk.LSU-6B* that was detected in the present study is flanked by the SNP markers BobWhite_c344_125 (3,870,206 bp) and BS00029434_51 (4,518,534 bp), around 200 Mbp away from *Fhb2* and its overlapping QTLs. This suggested that *QFfdk.LSU-6B* is different and a new source of resistance not previously identified. *QFfdk.LSU-6B* provided resistance to FDK over multiple environments and was the only one identified when FDK was averaged over all environments, suggesting its stable expression across environments. The gene vegetative cell wall protein (*gp1*) linked to the QTL has been shown to be associated with an FHB resistance QTL linked to Xwmc238-4B [72]. Another linked gene, 7-deoxyloganetic acid glucosyltransferase (*7-DLGT*), does not have a known role in disease resistance in plants. However, the presence of genes, such as protein disulfide isomerase that is induced in response to wheat blotch disease [73] and disease responsive gliadin genes at the locus, implies the involvement of the QTL in FHB resistance. 

In summary, our results indicated that multiple QTLs with individual minor effects control FHB resistance in wheat variety AGS 2060. However, the QTLs accounted for part of the total phenotypic variation for FHB resistance, which implied the opportunity for mapping additional QTLs with the use of new markers, closing the gaps in linkage map, especially in the D chromosomes, where few polymorphic markers were detected and mapped. The results also demonstrated that the susceptible variety AGS 2035 contributed some alleles for resistance under certain environments, such as Newport, Arkansas. A recent study [74] reported that multiple minor effect QTLs were responsible for FHB resistance in a wheat landrace Haiyanzhong and that two QTLs were derived from the susceptible variety Wheaton. AGS 2060 contained several previously identified QTLs. Some of the QTLs, such as *QFd&f.LSU-2A* and *QFd&f.LSU-7A,* provided broad-spectrum resistance to both DON and FDK over several environments and could prove to be valuable sources of resistance. WN19 was the most informative location, where nine of the 13 QTLs were detected. Three consistently expressed QTLs—*QFdon.LSU-5A*, *QFfdk.LSU-6B*, and *QFd&f.LSU-7A*—could potentially be newly identified sources of resistance to FHB in soft red winter wheat.

The large diversity in small effect QTLs demonstrates the difficulty in breeding for FHB resistance and partially explains the slow progress in varietal improvement. However, diversity also provides breeders with multiple sources to choose for improving FHB resistance in their elite material. Candidate genes, such as WD-repeat containing protein and LRR receptor-like serine-threonine protein kinase in linkage disequilibrium with the consistently expressed and potentially new QTLs, such as *QFd&f.LSU-7A,* will be validated in different test populations, and diagnostic KASP markers will be developed [28,29] for their use in marker-assisted selection for FHB resistance. The diagnostic KASP markers can be used to choose parents for crossing with complimentarily resistant QTLs to pyramid several resistant QTLs in advanced lines or to enrich early generation populations for QTLs of interest and to introgress new QTLs in the breeding program.

## Figures and Tables

**Table 1:** Descriptive statistics for FHB resistance ratings of AGS 2060 x AGS 2035 DH population at Baton Rouge 2017.

Trait	AGS 2060 Mean	AGS 2035 Mean	Population Mean	Population Range	Pr > F ^a^	C.V.	LSD (0.10)
DON (µg/g)	5.1	14.3	10.1	1.1–33.9	<0.0001	40.6	6.8
FDK %	19.2	36.7	26.1	5–70	<0.0001	37	16.2
Index %	21.5	30.3	27.2	3–80	0.0179	41.1	18.6
Incidence %	68.3	83.3	80	30–100	<0.0001	15.6	20.7
Severity %	31.7	36.7	33.3	10–80	<0.0001	35.7	19.8
FHB (0–9)	6.3	7.2	6.5	1–9	0.0008	21.1	2.1

^a^ Significant difference among the DH lines based on the F test from the ANOVA. DON = deoxynivalenol, FDK = *Fusarium* damaged kernel, FHB = *Fusarium* head blight, DH = doubled haploid.

**Table 2:** Descriptive statistics for FHB resistance ratings of AGS 2060 x AGS 2035 DH population at Baton Rouge 2018.

Trait	AGS 2060 Mean	AGS 2035 Mean	Population Mean	Population Range	Pr > F ^a^	C.V.	LSD (0.10)
DON (µg/g)	10.5	15.4	11.3	2.6–51.5	<0.0001	36.4	8.1
FDK %	19.2	43.3	33.7	5–70	<0.0001	21.9	14.6
Index %	18	42.8	28.9	3–80	<0.0001	26.2	15.1
Incidence %	56.7	82.8	74.9	20–100	<0.0001	11.4	16.8
Severity %	31.7	46.7	37.5	10–80	<0.0001	22.7	18.5
FHB (0–9)	4.5	8.5	6.9	2–9	<0.0001	13.6	1.4

^a^ Significant difference among the DH lines based on the F test from the ANOVA.

**Table 3:** Descriptive statistics for FHB resistance ratings of AGS 2060 x AGS 2035 DH population at Winnsboro 2018.

Trait	AGS 2060 Mean	AGS 2035 Mean	Population Mean	Population Range	Pr > F ^a^	C.V.	LSD (0.10)
DON (µg/g)	9.1	10.6	11.4	1.0–54.2	<0.0001	40.8	9.2
FDK %	15.8	39.2	30.1	5–65	<0.0001	20.4	12.1
Index %	13.3	38.3	26.8	8–54	<0.0001	36.4	14.9
Incidence %	48.3	84.1	68.5	30–90	<0.0001	16.8	17.7
Severity %	26.7	45	38.6	20–60	<0.0001	27.5	17.9
FHB (0–9)	4.8	8.1	6.8	3–9	<0.0001	16.8	1.5

^a^ Significant difference among the DH lines based on the F test from the ANOVA.

**Table 4:** Descriptive statistics for FHB resistance ratings of AGS 2060 x AGS 2035 DH population at Winnsboro 2019.

Trait	AGS 2060 Mean	AGS 2035 Mean	Population Mean	Population Range	Pr > F ^a^	C.V.	LSD (0.10)
DON (µg/g)	8.2	6.2	12.6	0.76–77.0	<0.0001	49.7	10.3
FDK %	41.7	60.8	55.9	25–85	<0.0001	15.5	14.3
Index %	18.5	24	23.6	6–81	<0.0001	24.9	9.8
Incidence %	61.7	71.7	67.7	30–100	<0.0001	10.9	12.3
Severity %	30.0	35.0	34.1	20–90	<0.0001	17.5	10.1
FHB (0–9)	6	7.2	6.7	3–9	<0.0001	9.6	1.1

^a^ Significant difference among the DH mapping population based on the F test from the ANOVA.

**Table 5:** Descriptive statistics for FHB resistance ratings of AGS 2060 x AGS 2035 DH population at Newport, 2018.

Trait	AGS 2060 Mean	AGS 2035 Mean	Population Mean	Population Range
DON (µg/g)	18.4	15.6	27.0	4.5–70.1
FDK %	30	40	43.8	15–80
Index %	31.7	45	35.1	5–100

**Table 6:** QTLs (quantitative trait loci) associated with resistance to DON and/or FDK under different test environments in Louisiana and Arkansas.

Trait	QTL Name	Environment ^a^	Flanking Markers	Position cM (Mbp)	LOD	PVE (%)	Add
**DON**	*QFdon.LSU-1A*	WN 2019	CAP7_c4833_141-wsnp_c24686_33942264	0.2 (25.5–66.8)	2.69	1.23	7.14
	*QFdon.LSU-1D*	BR 2018	Excalibur_c53900_86-Ra_c3045_2659	91.10 (419.7–420.6)	3.76	0.57	5.45
	*QFdon.LSU-5A*	WN 2018	Ra_c10762_1137-BS00023008_51	40.68 (8.1–8.2)	2.73	3.73	6.08
		WN Average	Ra_c10762_1137-BS00023008_51	41.19 (8.1–8.2)	3.80	5.89	7.06
		WN 2019	Ra_c10762_1137-BS00023008_51	41.19 (8.1–8.2)	7.82	1.74	11.62
	*QFdon.LSU-5B*	WN 2019	BS00079166_51-CAP11_c7700_247	210.8 (53.2–70.5)	4.4	1.71	−10.35
		BR 2018	BS00108020_51-IACX4548	203.2 (27.9–43.5)	3.82	0.61	−5.97
	*QFdon.LSU-6A*	WN 2018	wsnp_CAP11_c1137_6650-BS00023020_51	300.9 (542.2–560.8)	2.86	4.3	−3.69
		ARK 2018	CAP7_c4283_67-CAP11_c7092_120	309.2 (581.7–603.1)	2.55	1.81	−3.65
		WN 2019	CAP7_c4283_67-CAP11_c7092_120	309.4 (581.7–603.1)	4.4	1.82	9.20
	*QFdon.LSU-7B*	NP 2018	RAC875_c7251_656-Kukri_rep_c79716_729	23.3 (604.1–718.5)	3.16	3.45	8.09
		WN 2019	Kukri_rep_c79716_729-Tdurum_contig47854_142	25.8 (162.7–718.5)	4.69	1.52	10.83
**FDK**	*QFfdk.LSU-5A*	NP 2018	tplb0044j06_689-Ku_c12469_837	214.01 (596.1–596.5)	3.2	2.39	10.2
		WN 2019	BS00062907_51-Tdurum_contig11173_79	205.02 (33.01–35.3)	3.37	2.75	4.7
	*QFfdk.LSU-5B*	BR Average	BS00108020_51-ACX4548	203.30 (279.9–435.2)	3.99	1.83	−4.54
		BR 2018	BS00108020_51-IACX4548	203.21 (279.9–435.2)	3.76	0.57	−5.97
	*QFfdk.LSU-6B*	WN Average	BS00029434_51-BobWhite_c344_125	90.18 (3.9–4.5)	2.51	1.98	5.21
		Overall Average	BS00029434_51-BobWhite_c344_125	90.29 (3.9–4.5)	2.55	2.28	4.62
		NP 2018	BobWhite_c344_125BS00055174_51	95.29 (3.9–6.0)	3.77	0.58	5.81
**DON and FDK**	*QFd&f.LSU-1B*	WN 2019 (FDK)	Excalibur_c21451_352-BS00064162_51	145.99 (540.8–601)	4.82	1.41	−9.93
		WN Average (FDK)	BS00064162_51-Kukri_c9752_793	147.19 (581.2–601)	2.58	1.44	−8.19
		WN 2019 (DON)	BS00064162_51-Kukri_c9752_793	147.19 (581.2–601)	2.69	2.66	−5.22
	*QFd&f.LSU-2A*	WN 2019 (DON)	BS00067792_51-BS00023052_51	32.61 (3.1–319)	3.61	1.38	−8.61
		WN Average (DON)	BS00067792_51-BS00023052_51	32.61 (3.1–319)	5.54	2.8	−6.02
		NP 2018 (DON)	BS00067792_51-BS00023052_51	32.61 (3.1–319)	3.45	4.02	−8.89
		Overall (DON)	BS00067792_51-BS00023052_51	32.61 (3.1–319)	3.30	4.12	−3.80
		WN 2019 (FDK)	BS00067792_51-BS00023052_51	32.61 (3.1–319)	2.73	1.78	−9.83
	*QFd&f.LSU-2B*	BR 2017 (FDK)	RAC875_c35399_497-BobWhite_c15453_678	153.30 (740.8–747.6)	4.29	3.18	9.29
		BR 2017 (DON)	RAC875_c35399_497-BobWhite_c15453_679	153.30 (740.8–747.6)	3.44	1.57	3.39
		WN 2018 (FDK)	RAC875_c35399_497-BobWhite_c15453_679	153.60 (740.8–747.6)	2.59	4.46	8.19
		BR Average (DON)	RAC875_c35399_497-BobWhite_c15453_679	153.20 (740.8–747.6)	2.51	2.35	3.16
	*QFd&f.LSU-7A*	WN 2019 (DON)	IAAV822-Tdurum_contig42424_291	23.0 (1.4–5.4)	8.85	1.37	11.08
		WN 2019 (FDK)	IAAV822-Tdurum_contig42424_291	59.1 (1.4–5.4)	4.94	2.53	10.57
		WN 2018 (DON)	IAAV822-Tdurum_contig42424_291	23.9 (1.4–5.4)	2.93	2.99	5.32
		BR 2017 (DON)	IAAV822-Tdurum_contig42424_291	30.9 (1.4–5.4)	2.57	0.89	4.03
		BR 2017 (FDK)	IAAV822-Tdurum_contig42424_291	30.1 (1.4–5.4)	2.59	1.9	9.99

^a^ Environment is the combination of a location and year. BR = Baton Rouge, WN = Winnsboro, NP = Newport, cM = centiMorgan, LOD = logarithm of the odds, Mbp = megabase pairs, PVE = phenotypic variance explained, Add = additive effect.

## Supplementary Materials

The following are available online at https://www.mdpi.com/2073-4425/11/6/699/s1, Figure S1: Genetic map of wheat showing QTLs detected for DON and/or FDK. Marker intervals in cM are on the left of the chromosome while marker names are on the right. Colored lines indicate the trait evaluated for the QTL analysis. Table S1: Pearson correlation coefficients between FHB rating and days to heading in AGS2060 × AGS2035 DH population from data collected in Baton Rouge 2017. Table S2: Pearson correlation coefficients between FHB rating and days to heading in AGS2060 × AGS2035 DH population from data collected in Baton Rouge 2018. Table S3: Pearson correlation coefficients between FHB rating and relative maturity in AGS 2060 × AGS 2035 DH population from data collected in Winnsboro 2018. Table S4: Pearson correlation coefficients between FHB rating and relative maturity in AGS 2060 × AGS 2035 DH population from data collected in Winnsboro 2019. Table S5: Pearson correlation coefficients between FHB rating and days to heading in AGS 2060 × AGS 2035 DH population from data collected in Newport 2018. Table S6: Broad-sense heritability (*H_2_*) estimates for FDK and DON over environments. Table S7: Linkage map of the DH population developed by the SNP markers. Table S8: Average DON and FDK of the DH lines derived from AGS 2035 × AGS 2060. Table S9: Genes within 20 kb flanking the markers closest to the QTL peaks.
