# Supplementary material for: Identification of QTLs for Resistance to Fusarium Head Blight Using a Doubled Haploid Population Derived from Southeastern United States Soft Red Winter Wheat Varieties AGS 2060 and AGS 2035
Source: Genes (Basel). 2020 Jun 25;11(6):699. doi: 10.3390/genes11060699 (PMC7349885; doi:10.3390/genes11060699)

Supplementary Files

Table S1. Pearson correlation coefficients between FHB rating and days to heading in AGS2060 x AGS2035 DH population from data collected in Baton Rouge 2017.

	Days to Heading	Incidence	Severity	Index	FHB 0-9	FDK
Incidence	0.197 (<.0001)					
Severity	0.028 (0.567)	0.286 (<.0001)				
Index	0.089 (0.078)	0.557 (<.0001)	0.941 (<.0001)			
FHB 0-9	0.236 (<.0001)	0.802 (<.0001)	0.602 (<.0001)	0.756 (<.0001)		
FDK	-0.238 (<.0001)	0.099 (0.049)	0.336 (<.0001)	0.324 (<.0001)	0.252 (<.0001)	
DON	-0.170 (0.001)	0.135 (0.008)	0.369 (<.0001)	0.345 (<.0001)	0.237 (<.0001)	0.592 (<.0001)

Values in parenthesis indicate significance of correlation coefficients.

Table S2. Pearson correlation coefficients between FHB rating and days to heading in AGS2060 x AGS2035 DH population from data collected in Baton Rouge 2018.

	Days to heading	Incidence	Severity	Index	FHB 0-9	FDK
Incidence	-0.018 (0.722)					
Severity	0.282 (<.0001)	0.488 (<.0001)				
Index	0.182 (0.0003)	0.709 (<.0001)	0.949 (<.0001)			
FHB 0-9	0.218 (<.0001)	0.848 (<.0001)	0.723 (<.0001)	0.827 (<.0001)		
FDK	-0.107 (0.033)	0.548 (<.0001)	0.465 (<.0001)	0.563 (<.0001)	0.530 (<.0001)	
DON	-0.014 (0.780)	0.429 (<.0001)	0.519 (<.0001)	0.570 (<.0001)	0.464 (<.0001)	0.643 (<.0001)

Values in parenthesis indicate significance of correlation coefficients.

Table S3. Pearson correlation coefficients between FHB rating and relative maturity in AGS 2060 x AGS 2035 DH population from data collected in Winnsboro 2018.

	Rel Mat	Incidence	Severity	Index	FHB 0-9	FDK
Incidence	-0.132					
	(-0.063)					
Severity	0.234	0.511				
	(-0.001)	(<.0001)				
Index	0.084	0.8	0.913			
	(-0.239)	(<.0001)	(<.0001)			
FHB 0-9	-0.056	0.928	0.632	0.838		
	(-0.432)	(<.0001)	(<.0001)	(<.0001)		
FDK	0.078	0.581	0.513	0.622	0.626	
	(-0.269)	(<.0001)	(<.0001)	(<.0001)	(<.0001)	
DON	0.372	0.426	0.503	0.537	0.518	0.733
	(<.0001)	(<.0001)	(<.0001)	(<.0001)	(<.0001)	(<.0001)

Values in parenthesis indicate significance of correlation coefficients.

Table S4. Pearson correlation coefficients between FHB rating and relative maturity in AGS 2060 x AGS 2035 DH population from data collected in Winnsboro 2019.

	Rel Mat	Incidence	Severity	Index	FHB 0-9	FDK
Incidence	0.185					
	-0.0003					
Severity	0.249	0.532				
	(<.0001)	(<.0001)				
Index	0.249	0.795	0.923			
	(<.0001)	(<.0001)	(<.0001)			
FHB 0-9	0.235	0.876	0.659	0.802		
	(<.0001)	(<.0001)	(<.0001)	(<.0001)		
FDK	0.496	0.439	0.039	0.458	0.44	
	(<.0001)	(<.0001)	(<.0001)	(<.0001)	(<.0001)	
DON	0.697	0.233	0.307	0.319	0.241	0.53175
	(<.0001)	(<.0001)	(<.0001)	(<.0001)	(<.0001)	(<.0001)

Values in parenthesis indicate significance of correlation coefficients.

Table S5. Pearson correlation coefficients between FHB rating and days to heading in AGS 2060 x AGS 2035 DH population from data collected in Newport 2018.

	Index	Days to heading	FDK
Days to heading	-0.288		
	(<.0001)		
FDK	0.409	-0.155	
	(<.0001)	0.028	
DON	0.038	0.306	0.547
	-0.0595	(<.0001)	(<.0001)

Values in parenthesis indicate significance of correlation coefficients.

Supplementary Table S6. Broad-sense heritability (H^2) estimates for FDK and DON over environments

Over environments

Trait	Environment	Source	DF	SS	MS	F-value	Pr>F	
FDK	BR	Model	198	52376.43	264.5274	7.9188	0.00E+00	
FDK	BR	Error	197	6580.821	33.4052			
FDK	BR	Total	395	58957.25				
FDK	BR	R-square(%)	88.838					
FDK	BR	Source	DF	SS	MS	F-value	Pr>F	EstimatedVar
FDK	BR	Block	1	3.5511	3.5511	0.1063	7.45E-01	0
FDK	BR	Genotype	197	52372.88	265.8522	7.9584	0.00E+00	116.2235
FDK	BR	LSD(0.05)	11.3981					
FDK	BR	LSD(0.01)	15.0332					
FDK	BR	H^2_per_plot	0.7767					
FDK	BR	H^2_per_mean	0.8743					

Trait	Environment	Source	DF	SS	MS	F-value	Pr>F	
FDK	WN	Model	198	44569.36	225.0978	7.4312	0.00E+00	
FDK	WN	Error	197	5967.315	30.2909			
FDK	WN	Total	395	50536.68				
FDK	WN	R-square(%)	88.1921					
FDK	WN	Source	DF	SS	MS	F-value	Pr>F	EstimatedVar
FDK	WN	Block	1	4.5613	4.5613	0.1506	6.98E-01	0
FDK	WN	Genotype	197	44564.8	226.2172	7.4681	0.00E+00	97.9632
FDK	WN	LSD(0.05)	10.8538					
FDK	WN	LSD(0.01)	14.3153					
FDK	WN	H^2_per_plot	0.7638					
FDK	WN	H^2_per_mean	0.8661					

Trait	Environment	Source	DF	SS	MS	F-value	Pr>F	
DON	BR	Model	198	8880.531	44.8512	4.7593	0.00E+00	
DON	BR	Error	197	1856.493	9.4238			
DON	BR	Total	395	10737.02				
DON	BR	R-square(%)	82.7094					
DON	BR	Source	DF	SS	MS	F-value	Pr>F	EstimatedVar
DON	BR	Block	1	500.3218	500.3218	53.0912	0.00E+00	2.4793
DON	BR	Genotype	197	8380.209	42.5391	4.514	0.00E+00	16.5577
DON	BR	LSD(0.05)	6.0539					
DON	BR	LSD(0.01)	7.9847					
DON	BR	H^2_per_plot	0.6373					
DON	BR	H^2_per_mean	0.7785					

Trait	Environment	Source	DF	SS	MS	F-value	Pr>F	
DON	WN	Model	198	19965.33	100.835	5.4181	0.00E+00	
DON	WN	Error	197	3666.336	18.6108			
DON	WN	Total	395	23631.66				
DON	WN	R-square(%)	84.4855					
DON	WN	Source	DF	SS	MS	F-value	Pr>F	EstimatedVar
DON	WN	Block	1	142.6376	142.6376	7.6642	6.17E-03	0.6264
DON	WN	Genotype	197	19822.69	100.6228	5.4067	0.00E+00	41.006
DON	WN	LSD(0.05)	8.5076					
DON	WN	LSD(0.01)	11.2209					
DON	WN	H^2_per_plot	0.6878					
DON	WN	H^2_per_mean	0.815					

Trait	Environment	Source	DF	SS	MS	F-value	Pr>F	
FDK	Combined	Model	397	131548.5	331.3565	10.4043	0.00E+00	
FDK	Combined	Error	394	12548.17	31.8481			
FDK	Combined	Total	791	144096.7				
FDK	Combined	R-square(%)	91.2918					
FDK	Combined	Source	DF	SS	MS	F-value	Pr>F	EstimatedVar
FDK	Combined	Block/Env	2	8.1125	4.0562	0.1274	8.80E-01	0
FDK	Combined	Genotype	197	78918.59	400.602	12.5785	0.00E+00	92.1885
FDK	Combined	Environment	1	34602.49	34602.49	1086.484	0.00E+00	87.2996
FDK	Combined	GE_interaction	197	18019.34	91.4687	2.872	0.00E+00	29.8103
FDK	Combined	LSD(0.05)	13.2955					
FDK	Combined	LSD(0.01)	17.5044					
FDK	Combined	H^2_per_plot	0.5992					
FDK	Combined	H^2_per_mean	0.8013					
FDK	Homogeneity	BartlettTest	0.4702	4.93E-01				

Trait	Environment	Source	DF	SS	MS	F-value	Pr>F	
DON	Combined	Model	397	29226.56	73.6185	5.252	0.00E+00	
DON	Combined	Error	394	5522.826	14.0173			
DON	Combined	Total	791	34749.39				
DON	Combined	R-square(%)	84.1067					
DON	Combined	Source	DF	SS	MS	F-value	Pr>F	EstimatedVar
DON	Combined	Block/Env	2	642.9496	321.4748	22.9341	0.00E+00	1.5528
DON	Combined	Genotype	197	16685.99	84.7005	6.0426	0.00E+00	17.6708
DON	Combined	Environment	1	380.7129	380.7129	27.1602	0.00E+00	0.926
DON	Combined	GE_interaction	197	11516.91	58.4615	4.1707	0.00E+00	22.2221
DON	Combined	LSD(0.05)	10.6293					
DON	Combined	LSD(0.01)	13.9941					
DON	Combined	H^2_per_plot	0.3278					
DON	Combined	H^2_per_mean	0.5473					

DON	Homogeneity	BartlettTest	22.3233	3.34E-06

Over years

Trait	Environment	Source	DF	SS	MS	F-value	Pr>F	
FDK	17	Model	198	55028.3	277.9207	2.9626	9.72E-06	
FDK	17	Error	193	18105.12	93.8089			
FDK	17	Total	391	73133.42				
FDK	17	R-square(%)	75.2437					
FDK	17	Source	DF	SS	MS	F-value	Pr>F	EstimatedVar
FDK	17	Block	1	13.3354	13.3354	0.1422	7.07E-01	0
FDK	17	Genotype	197	55014.97	279.2638	2.9769	5.72E-06	93.6736
FDK	17	LSD(0.05)	19.2002					
FDK	17	LSD(0.01)	25.3255					
FDK	17	H^2_per_plot	0.4996					
FDK	17	H^2_per_mean	0.6641					

Trait	Environment	Source	DF	SS	MS	F-value	Pr>F	
FDK	18	Model	198	60498.01	305.5455	12.7941	0.00E+00	
FDK	18	Error	197	4704.689	23.8817			
FDK	18	Total	395	65202.7				
FDK	18	R-square(%)	92.7845					
FDK	18	Source	DF	SS	MS	F-value	Pr>F	EstimatedVar
FDK	18	Block	1	17.1875	17.1875	0.7197	3.97E-01	0
FDK	18	Genotype	197	60480.82	307.0092	12.8554	0.00E+00	141.5638
FDK	18	LSD(0.05)	9.6373					
FDK	18	LSD(0.01)	12.7109					
FDK	18	H^2_per_plot	0.8557					
FDK	18	H^2_per_mean	0.9222					

Trait	Environment	Source	DF	SS	MS	F-value	Pr>F	
DON	17	Model	198	9560.827	48.287	2.8686	1.04E-05	
DON	17	Error	193	3248.8	16.8332			
DON	17	Total	391	12809.63				
DON	17	R-square(%)	74.6378					
DON	17	Source	DF	SS	MS	F-value	Pr>F	EstimatedVar
DON	17	Block	1	572.3271	572.3271	34	2.62E-06	2.8342
DON	17	Genotype	197	8988.5	45.6269	2.7105	8.70E-06	14.5438
DON	17	LSD(0.05)	8.1333					
DON	17	LSD(0.01)	10.728					
DON	17	H^2_per_plot	0.4635					
DON	17	H^2_per_mean	0.6311					

Trait	Environment	Source	DF	SS	MS	F-value	Pr>F
DON	18	Model	198	10919.3	55.148	5.9108	0.00E+00

DON	18	Error	197	1838.026	9.3301			
DON	18	Total	395	12757.33				
DON	18	R-square(%)	85.5924					
DON	18	Source	DF	SS	MS	F-value	Pr>F	EstimatedVar
DON	18	Block	1	137.6238	137.6238	14.7505	1.68E-04	0.6479
DON	18	Genotype	197	10781.67	54.7293	5.8659	0.00E+00	22.6996
DON	18	LSD(0.05)	6.0238					
DON	18	LSD(0.01)	7.9449					
DON	18	H^2_per_plot	0.7087					
DON	18	H^2_per_mean	0.8295					

Trait	Environment	Source	DF	SS	MS	F-value	Pr>F	
FDK	Combined	Model	397	121733.5	306.6336	5.2428	0.00E+00	
FDK	Combined	Error	390	22809.88	58.4869			
FDK	Combined	Total	787	144543.4				
FDK	Combined	R-square(%)	84.2194					
FDK	Combined	Source	DF	SS	MS	F-value	Pr>F	EstimatedVar
FDK	Combined	Block/Env	2	30.4679	15.234	0.2605	7.71E-01	0
FDK	Combined	Genotype	197	91859.15	466.2901	7.9726	0.00E+00	102.4683
FDK	Combined	Environment	1	6196.534	6196.534	105.9474	0.00E+00	15.5788
FDK	Combined	GE_interaction	197	23647.38	120.0375	2.0524	0.00E+00	30.9315
FDK	Combined	LSD(0.05)	15.2899					
FDK	Combined	LSD(0.01)	20.1305					
FDK	Combined	H^2_per_plot	0.534					
FDK	Combined	H^2_per_mean	0.7726					
FDK	Homogeneity	BartlettTest	85.0476	0.00E+00				

Trait	Environment	Source	DF	SS	MS	F-value	Pr>F	
DON	Combined	Model	397	20769.42	52.3159	4.011	0.00E+00	
DON	Combined	Error	390	5086.828	13.0431			
DON	Combined	Total	787	25856.25				
DON	Combined	R-square(%)	80.3265					
DON	Combined	Source	DF	SS	MS	F-value	Pr>F	EstimatedVar
DON	Combined	Block/Env	2	710.1093	355.0546	27.2215	1.61E-06	1.7361
DON	Combined	Genotype	197	15238.57	77.3531	5.9306	0.00E+00	16.1591
DON	Combined	Environment	1	288.8243	288.8243	22.1438	4.89E-06	0.7
DON	Combined	GE_interaction	197	4531.924	23.0047	1.7637	1.19E-06	5.0061
DON	Combined	LSD(0.05)	6.6922					
DON	Combined	LSD(0.01)	8.8108					
DON	Combined	H^2_per_plot	0.4724					
DON	Combined	H^2_per_mean	0.7365					
DON	Homogeneity	BartlettTest	16.7238	4.49E-05				

Table S7. Linkage map of the DH population developed by the SNP markers

Chromosome	Marker #	Coverage (cM)	Avg. distance/marker
1A	109	141.17	1.30
1B	147	160.11	1.09
1D	54	153.66	2.85
2A	116	225.33	1.94
2B	367	168.42	0.46
2D	69	166.12	2.41
3A	91	175.57	1.93
3B	126	179.83	1.43
3D	6	77.92	12.99
4A	64	132.48	2.07
4B	96	170.36	1.77
4D	17	85.14	5.01
5A	92	254.71	2.77
5B	161	215.28	1.34
5D	14	129.79	9.27
6A	114	310.73	2.73
6B	112	172.39	1.54
6D	26	212.03	8.16
7A	103	230.94	2.24
7B	108	177.15	1.64
7D	18	207.67	11.54
	Total = 2,011	Total = 3746.8	Average = 1.86

Table S8. Average DON and FDK of the DH lines derived from AGS 2035 x AGS 2060

	Trait	
	**FDK**	**DON**
AGS2035-1	44.4	11.8
AGS2060-1	24.4	9.5
LA12016DHA-1	58.1	23.9
LA12016DHA-10	46.7	20.2
LA12016DHA-100	37.8	11.6
LA12016DHA-101	31.1	8.2
LA12016DHA-102	49.4	17.8
LA12016DHA-104	30.6	9.3
LA12016DHA-106	33.9	10.2
LA12016DHA-107	30.0	8.6
LA12016DHA-108	32.2	9.7
LA12016DHA-11	24.4	6.5
LA12016DHA-12	33.9	5.4
LA12016DHA-13	41.1	12.6
LA12016DHA-14	57.2	13.3
LA12016DHA-15	26.1	25.2
LA12016DHA-16	52.8	15.4
LA12016DHA-17	35.0	9.0
LA12016DHA-18	30.6	9.6
LA12016DHA-19	54.4	18.9
LA12016DHA-2	34.4	10.7
LA12016DHA-20	37.2	13.1
LA12016DHA-21	30.6	6.7
LA12016DHA-22	45.0	13.8
LA12016DHA-23	37.2	8.3
LA12016DHA-24	48.9	13.6
LA12016DHA-25	31.1	7.9
LA12016DHA-26	45.6	21.0
LA12016DHA-27	51.1	16.3
LA12016DHA-28	27.8	8.6
LA12016DHA-29	26.1	7.7
LA12016DHA-3	52.8	16.4
LA12016DHA-30	42.8	11.0
LA12016DHA-31	34.4	10.5
LA12016DHA-32	35.6	9.6
LA12016DHA-33	39.4	13.6
LA12016DHA-34	40.0	19.1
LA12016DHA-35	43.9	10.9
LA12016DHA-36	21.7	7.4
LA12016DHA-37	38.9	23.7
LA12016DHA-38	22.2	9.1
LA12016DHA-39	20.6	16.1
LA12016DHA-4	23.9	9.7

LA12016DHA-40	18.3	11.5
LA12016DHA-41	27.2	6.5
LA12016DHA-42	32.8	15.5
LA12016DHA-43	16.7	9.6
LA12016DHA-44	50.6	17.5
LA12016DHA-45	45.6	14.0
LA12016DHA-46	42.8	13.2
LA12016DHA-47	41.1	22.2
LA12016DHA-48	27.2	6.2
LA12016DHA-49	26.1	8.2
LA12016DHA-5	56.1	19.4
LA12016DHA-50	24.4	9.2
LA12016DHA-51	47.8	18.0
LA12016DHA-52	33.3	13.0
LA12016DHA-53	31.1	8.8
LA12016DHA-54	22.8	10.6
LA12016DHA-55	46.7	19.9
LA12016DHA-56	57.2	24.1
LA12016DHA-57	17.8	6.6
LA12016DHA-58	46.1	13.2
LA12016DHA-59	32.8	12.2
LA12016DHA-6	22.8	12.0
LA12016DHA-60	28.9	8.4
LA12016DHA-61	37.2	10.4
LA12016DHA-62	47.8	16.1
LA12016DHA-63	33.3	14.4
LA12016DHA-64	31.1	9.9
LA12016DHA-65	43.9	9.8
LA12016DHA-66	32.2	10.9
LA12016DHA-67	32.2	17.0
LA12016DHA-68	35.0	11.5
LA12016DHA-69	56.7	15.3
LA12016DHA-7	18.3	6.7
LA12016DHA-70	33.9	9.8
LA12016DHA-71	46.1	18.1
LA12016DHA-72	36.7	11.4
LA12016DHA-73	38.3	11.5
LA12016DHA-74	23.9	11.0
LA12016DHA-75	17.8	7.7
LA12016DHA-76	27.8	10.4
LA12016DHA-77	28.3	21.4
LA12016DHA-78	38.9	21.6
LA12016DHA-79	20.6	4.5
LA12016DHA-8	38.9	11.7
LA12016DHA-80	43.9	13.3
LA12016DHA-81	43.3	11.6

LA12016DHA-82	43.9	11.3
LA12016DHA-83	26.7	13.9
LA12016DHA-84	50.0	16.0
LA12016DHA-85	46.7	14.4
LA12016DHA-86	30.6	15.0
LA12016DHA-87	56.1	14.4
LA12016DHA-88	57.8	28.0
LA12016DHA-89	45.6	17.3
LA12016DHA-9	29.4	8.2
LA12016DHA-90	25.6	15.0
LA12016DHA-91	35.0	12.4
LA12016DHA-92	25.6	10.1
LA12016DHA-93	34.4	17.1
LA12016DHA-94	52.2	16.8
LA12016DHA-95	26.7	8.0
LA12016DHA-96	40.6	14.2
LA12016DHA-97	45.0	13.5
LA12016DHA-99	33.9	12.8
LA12016DHB-1	45.6	17.6
LA12016DHB-10	31.1	7.2
LA12016DHB-11	30.0	6.2
LA12016DHB-12	40.6	15.9
LA12016DHB-13	60.6	27.3
LA12016DHB-14	29.4	19.4
LA12016DHB-15	23.3	20.5
LA12016DHB-16	23.9	5.0
LA12016DHB-17	43.8	17.2
LA12016DHB-19	28.3	11.7
LA12016DHB-2	41.1	11.9
LA12016DHB-20	52.8	26.7
LA12016DHB-21	38.9	10.7
LA12016DHB-22	50.0	22.7
LA12016DHB-23	38.9	6.7
LA12016DHB-24	43.9	13.5
LA12016DHB-25	41.1	13.0
LA12016DHB-26	41.7	12.7
LA12016DHB-27	43.9	17.0
LA12016DHB-28	56.7	18.5
LA12016DHB-29	20.0	4.9
LA12016DHB-3	31.1	11.0
LA12016DHB-30	21.7	4.7
LA12016DHB-31	41.1	11.6
LA12016DHB-32	52.2	20.9
LA12016DHB-33	36.7	11.8
LA12016DHB-34	43.9	16.5
LA12016DHB-35	31.1	10.7

LA12016DHB-36	25.6	7.7
LA12016DHB-37	40.6	20.0
LA12016DHB-38	45.0	13.3
LA12016DHB-39	56.7	17.6
LA12016DHB-4	36.7	18.9
LA12016DHB-40	31.3	16.8
LA12016DHB-41	34.4	9.1
LA12016DHB-42	26.1	9.1
LA12016DHB-43	33.3	12.0
LA12016DHB-44	48.8	10.2
LA12016DHB-45	57.8	19.1
LA12016DHB-46	27.8	12.6
LA12016DHB-47	44.4	19.1
LA12016DHB-48	36.1	13.0
LA12016DHB-49	46.1	12.2
LA12016DHB-5	38.3	28.6
LA12016DHB-50	44.4	17.9
LA12016DHB-51	26.1	6.7
LA12016DHB-52	54.4	15.7
LA12016DHB-53	33.9	12.5
LA12016DHB-54	43.3	8.0
LA12016DHB-55	52.8	17.9
LA12016DHB-56	38.3	10.3
LA12016DHB-57	32.2	10.4
LA12016DHB-58	38.9	15.9
LA12016DHB-59	21.7	5.2
LA12016DHB-6	28.3	7.3
LA12016DHB-60	35.0	16.5
LA12016DHB-62	50.0	10.1
LA12016DHB-63	39.4	12.8
LA12016DHB-64	50.0	12.7
LA12016DHB-65	39.4	14.2
LA12016DHB-66	42.2	13.2
LA12016DHB-67	44.4	19.1
LA12016DHB-68	37.8	9.4
LA12016DHB-69	52.2	14.4
LA12016DHB-7	39.4	16.5
LA12016DHB-70	31.1	9.5
LA12016DHB-71	35.6	9.7
LA12016DHB-72	42.2	17.7
LA12016DHB-73	56.1	13.9
LA12016DHB-74	32.2	10.1
LA12016DHB-75	46.1	15.8
LA12016DHB-76	33.3	11.6
LA12016DHB-77	33.3	10.3
LA12016DHB-78	32.2	10.3

LA12016DHB-79	28.9	8.5
LA12016DHB-8	32.8	23.8
LA12016DHB-80	38.3	15.7
LA12016DHB-81	40.6	12.8
LA12016DHB-82	25.0	7.6
LA12016DHB-83	29.4	10.1
LA12016DHB-85	38.9	14.2
LA12016DHB-86	29.4	10.2
LA12016DHB-89	39.4	13.4
LA12016DHB-9	27.8	11.1
LA12016DHB-90	36.1	11.2
LA12016DHB-91	29.4	6.2
LA12016DHB-92	39.4	17.4

Table S9. Genes within 20 kb flanking the markers closest to the QTL peaks

**Marker (closest to QTL)**	**Genes similar to**
BS00067792_51	*Triticum aestivum* gamma gliadin-A1, gamma gliadin-A3, gamma gliadin-A4, and LMW-A2 genes
	Aegilops tauschii subsp. tauschii folate-biopterin transporter 1, chloroplastic-like (LOC109754426), transcript variant X2
	Triticum aestivum RAR1 (RAR1) gene, RAR1-A1 allele
BS00069019_51	*Aegilops tauschii* subsp. tauschii small RNA degrading nuclease 1-like (LOC109736362)
	miR1436-3p-117
	miR1118-3p-37
	miR1436-3p-31
	miR1118-3p-59
Tdurum_contig11173_79	*Aegilops tauschii* subsp. tauschii peptidyl-prolyl cis-trans isomerase NIMA-interacting 4 (LOC109773849)
	Aegilops tauschii subsp. tauschii fructose-bisphosphate aldolase-lysine N-methyltransferase, chloroplastic (LOC109773851)
	Aegilops tauschii subsp. tauschii pentatricopeptide repeat-containing protein At5g61400-like (LOC109773850)
BS00064162_51	Aegilops tauschii subsp. tauschii uncharacterized LOC109734195 (LOC109734195)
	Aegilops tauschii subsp. tauschii ATP-dependent DNA helicase PIF6-like (LOC109744440)
	Aegilops tauschii subsp. tauschii uncharacterized LOC109734195 (LOC109734195)
CAP11_c7700_247	*Aegilops tauschii* subsp. tauschii E3 ubiquitin-protein ligase RNF170-like (LOC109774652)
	Aegilops tauschii subsp. tauschii uncharacterized LOC109774651 (LOC109774651), transcript variant X2
	Aegilops tauschii subsp. tauschii uncharacterized LOC109774655 (LOC109774655)
	Thinopyrum elongatum early salt stress and cold acclimation-induced protein 2-1 (Esi2) gene
BobWhite_c344_125	*Aegilops tauschii* subsp. tauschii 7-deoxyloganetic acid glucosyltransferase-like (LOC109765156)
	Triticum aestivum omega gliadin-D1, omega gliadin-D2, omega gliadin-D3, gamma gliadin-D1, delta gliadin-D1, gamma gliadin-D2, gamma gliadin-D3, gamma gliadin-D4, LMW-D1, LMW-D2, LMW-D3, LMW-D6, and LMW-D7 genes
	Aegilops tauschii subsp. tauschii vegetative cell wall protein gp1-like (LOC109777230)
	Aegilops tauschii subsp. tauschii protein disulfide isomerase-like 1-4 (LOC109756626)

wsnp_CAP11_c1137_665073	*Triticum aestivum* geranylgeranyl hydrogenase mRNA
IAAV822	WD repeat-containing protein 89 homolog [*Aegilops tauschii subsp. tauschii*]
Tdurum_contig42424_291	LRR receptor-like serine/threonine-protein kinase HSL2 [*Triticum urartu*]

Figure S1. Genetic map of wheat showing QTLs detected for DON and/or FDK. Marker intervals in cM are on the left of the chromosome while marker names are on the right. Colored lines indicate the trait evaluated for the QTL analysis.

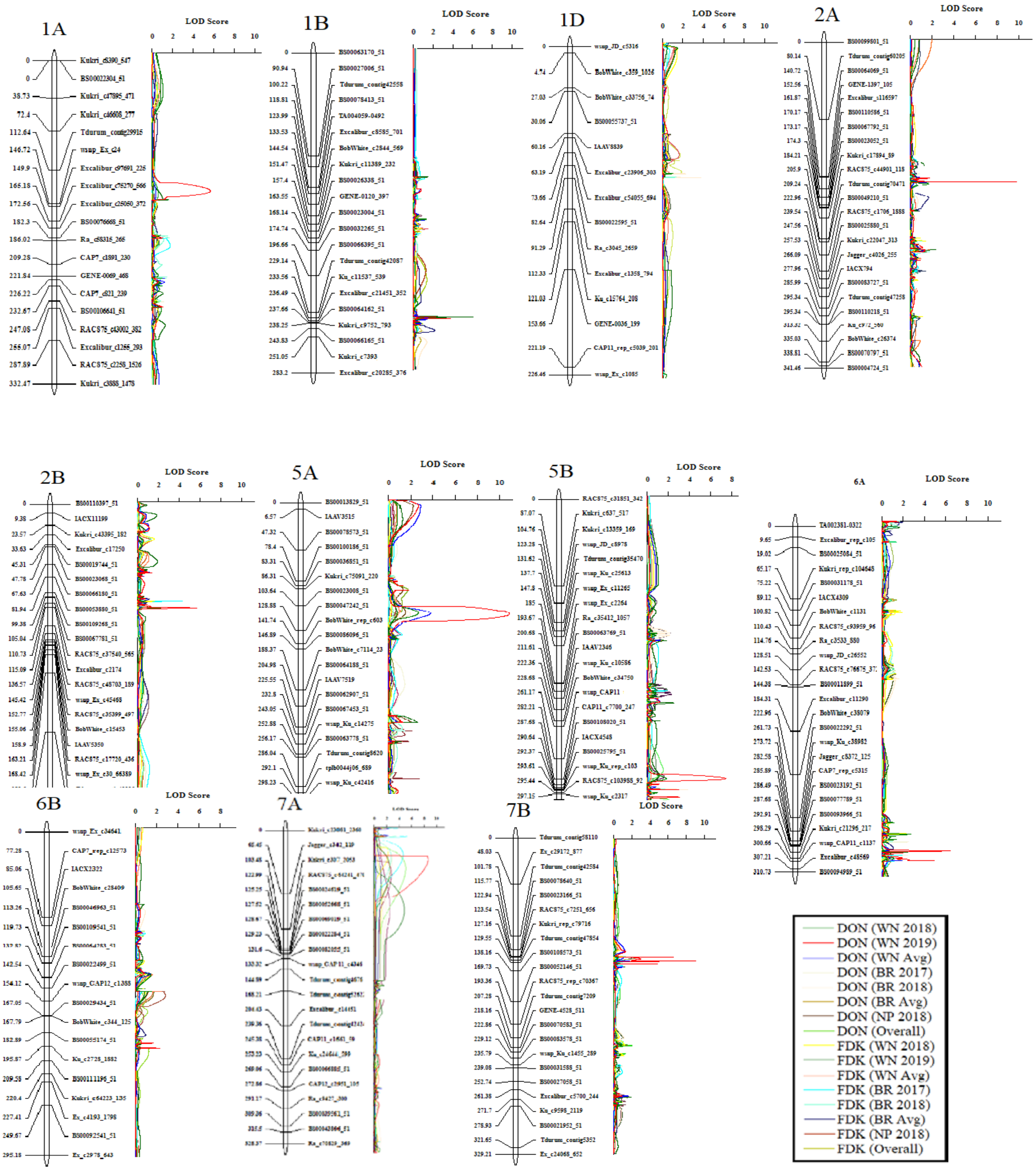

Supplement: Supplementary file 1 [file genes-11-00699-s001.pdf]
